# A hub of activity

**DOI:** 10.7554/eLife.93706

**Published:** 2023-11-29

**Authors:** Virginia L Pimmett, Mounia Lagha

**Affiliations:** 1 Institut de Génétique Moléculaire de Montpellier, University of Montpellier, CNRS UMR 5535 Paris France

**Keywords:** transcription hub, transcription factors, mastermind, notch, quantitative imaging, *Drosophila*, transcription kinetics, *D. melanogaster*

## Abstract

Imaging experiments reveal the complex and dynamic nature of the transcriptional hubs associated with Notch signaling.

**Related research article** deHaro-Arbona FJ, Roussos C, Baloul S, Townson J, Gomez-Lamarca MJ, Bray S. 2023. Dynamic modes of Notch transcription hubs conferring memory and stochastic activation revealed by live imaging the co-activator Mastermind. *eLife* **12**:RP92083. doi: 10.7554/eLife.92083.

It is exceedingly rare that a protein acts alone – more often, proteins co-operate with one another so that they can function with greater speed, specificity, or reactivity. The process by which they assemble at promoter regions within the genome in order to initiate gene transcription is relatively well understood. However, much less is known about how proteins come together at enhancer regions – sites that regulate gene expression – and how each protein contributes to transcription.

One form of co-operation involves transcription factors and other regulatory proteins physically associating with one another to form a ‘hub’ – a pocket of high protein concentration – around gene enhancers and promoters in the cell nucleus (Hnisz et al., 2017). But how do these hubs form and evolve over time? How do they ‘sense’ upstream signaling input? And how do they foster transcription? Now, in eLife, Sarah Bray from the University of Cambridge and colleagues – including Javier deHaro-Arbona as first author – report the results of experiments that will help to answer these questions (deHaro-Arbona et al., 2023; Figure 1).

**Figure 1.**
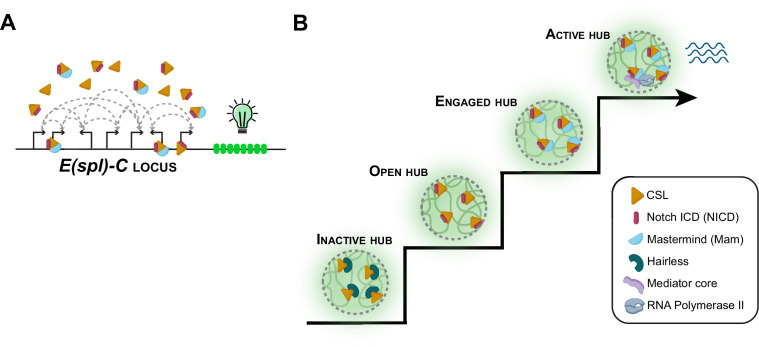
Notch signaling and transcriptional hubs. (**A**) The *E(spl)-C* locus contains multiple genes that are regulated by the Notch signaling pathway. (**B**) When there is no Notch signal, the transcriptional hub near the *E(spl)-C* locus is inactive (bottom left): an inactive hub represses gene expression because CSL is in complex with the suppressor protein Hairless. When there is a Notch signal, CSL associates with the Notch intracellular domain (NICD) to create an open hub, which targets Notch-activated genes, and the CSL-NICD complex recruits the co-activator Mastermind to create an engaged hub. A subset of engaged hubs become active (top right) by recruiting a Mediator complex and RNA polymerase II to engage in transcription.

Image credits: Figure created using elements from Biorender.com.

The team focused on the Notch signaling pathway, which becomes activated when a Notch receptor at the plasma membrane of a cell binds to its ligand. This triggers the release of the Notch intracellular domain (NICD) into the cell where it recruits two other proteins: a transcription factor known as CSL, and the co-activator protein Mastermind. Together, with RNA Polymerase II (the protein complex that transcribes DNA) and Mediator (a complex that is also involved in transcription), they regulate the expression of many genes.

To study this process, deHaro-Arbona et al. performed ex vivo imaging of *Drosophila* larval salivary glands. The experiments looked at a gene locus called *E(spl)-C* (short for the *Enhancer of split Complex*), which contains multiple genes that are regulated by Notch signaling. This locus and its regulators (CSL, Mastermind, RNA Polymerase II and Mediator) were each fluorescently labelled and monitored in live cells.

The findings show that Mastermind and CSL form a hub at the *E(spl)-C* locus when Notch signaling is activated. Intriguingly, the amount of CSL recruited to the hub did not correlate with the number of CSL binding sites at the locus, suggesting that CSL proteins do not interact with these regions in a one-to-one ratio. This more complex type of co-operation, known as non-stoichiometric binding, may be mediated by weak protein-protein interactions. As intrinsically disordered regions in proteins are often implicated in such interactions (Chong et al., 2018), deHaro-Arbona et al. investigated the role of these regions in NICD, CSL and Mastermind. They found that while the disordered region of NICD targeted the *E(spl)-C* locus, the disordered regions in CSL and Mastermind made only minor contributions to the hub. It appears, therefore, that the hub only partially relies on intrinsically disordered regions.

This is consistent with prior observations that not all hubs are equal and, instead, they consist of local microenvironments of various sizes, compositions and biophysical properties, which are generally dynamic and evolve during transcription (Sharp et al., 2022). Hubs are generally located in places where molecules rely on slower diffusion kinetics to search for their target (Lu and Lionnet, 2021). This is also the case for Notch activator complexes, which exhibited slow diffusion and a long residence time at *E(spl)-C*.

To examine how the function of the hub depends on its individual components, deHaro-Arbona et al. inhibited the recruitment of the Mastermind protein. This did not impact the recruitment of CSL to the hub. It also did not prevent Notch signalling from increasing chromatin accessibility, suggesting the hub does not need Mastermind in order to access chromatin. However, another protein in the hub, a Mediator complex named Med13, was not recruited. These findings indicate that while some changes induced by Notch signaling can occur independently of Mastermind, it is essential for recruitment of Med13.

Next, deHaro-Arbona et al. investigated how the Notch hubs impact transcription. Live imaging showed that only a third of hubs recruited the Mediator complex and RNA Polymerase II (which enable transcription), and only a third of cells showed active transcription following signaling. This is consistent with the view that even in the presence of all the necessary transcription factors, active transcription is probabilistic (Lu and Lionnet, 2021).

It remains an open question how promoters decode transcriptional hubs to trigger a specific choreography of RNA Polymerase II activation. A transcriptional hub being present does not always result in bursts of transcriptional activity (Dufourt et al., 2018; Mir et al., 2018). In some contexts, a high local density of newly synthesized transcripts can dissolve hubs (Cho and O’Farrell, 2022; Sharp et al., 2022), allowing hubs to self-limit their existence using RNA-mediated feedback mechanisms. The transient nature of hubs could be contributing to the stochasticity of transcription, but this hypothesis warrants more investigation.

Finally, deHaro-Arbona et al. asked if the action of a Notch transcription hub could persist once cells are no longer subject to a Notch input signal. Optogenetics experiments revealed that loci with prior exposure to Notch signaling were re-activated by subsequent Notch signaling more rapidly than naïve cells, thus displaying a form of ‘memory’. deHaro-Arbona et al. propose this memory might arise from the transcription factor CSL ‘book-marking’ the *E(spl)-C* locus during mitosis in order to speed up transcriptional reactivation, as has been observed in experiments with other transcription factors in vivo (Bellec et al., 2022; Gonzalez et al., 2021). This is clearly a topic for further research.

By revealing the dynamic nature of these transcriptional hubs (Figure 1), the work of deHaro-Arbona et al. leads to a number of questions. How long do hubs take to form at the target locus after signal induction, and how long do they take to dissolve once the signal stops? How does the hub evolve once transcription is activated, and how might that impact the timing and variability of transcriptional activity? Overall, the work enriches our understanding of hub formation and the role of hubs in modulating transcription, and provides a flexible platform to explore the function of transcriptional hubs in living organisms.
